# Circular RNA_0033596 aggravates endothelial cell injury induced by oxidized low-density lipoprotein via microRNA-217-5p /chloride intracellular channel 4 axis

**DOI:** 10.1080/21655979.2022.2027062

**Published:** 2022-01-26

**Authors:** Bai Jing, Zhou Hui

**Affiliations:** a Department of Cardiology, Renmin Hospital of Wuhan University, Wuhan, PR China; b Department of Ultrasound, wuhan Prevention and Treatment Center for Occupational Diseases Wuhan PR China

**Keywords:** Atherosclerosis, Circ_0033596, MiR-217-5p, CLIC4

## Abstract

In recent years, the modulatory functions of some circular RNAs (circRNAs) in the pathogenesis of atherosclerosis (AS) have been reported. Nonetheless, the role of circular RNA_0033596 (circ_0033596) in AS and its mechanism remains unclarified. In this study, oxidized low-density lipoprotein (ox-LDL) was applied to treat human umbilical vein endothelial cells (HUVECs) to establish a cell model of endothelial cell injury. Western blot and quantitative real-time polymerase chain reaction (qRT-PCR) were employed to detect the expression of circ_0033596, microRNA-217-5p (miR-217-5p), and chloride intracellular channel 4 (CLIC4) in HUVECs. The binding sites between circ_0033596 and miR-217-5p, as well as between miR-217-5p and CLIC4 mRNA 3ʹUTR were determined through a dual-luciferase reporter gene assay. It was found that circ_0033596 expression was increased in ox-LDL-induced HUVECs. After ox-LDL stimulation, HUVEC viability and cell cycle progression were inhibited, and the apoptosis was promoted, while circ_0033596 overexpression aggravated these effects. MiR-217-5p was identified as a downstream target of circ_0033596, and circ_0033596 negatively regulated miR-217-5p expression. CLIC4 was identified as miR-217-5p’s downstream target gene and could be positively modulated by circ_0033596. All in all circ_0033596 aggravates ox-LDL-induced HUVEC apoptosis by regulating the miR-217-5p/CLIC4 axis, by which circ_0033596 participates in the pathogenesis of AS.

## 1. Introduction

Known as a chronic inflammatory disease of the vascular wall, atherosclerosis (AS) contributes to the pathogenesis of a lot of cardiovascular diseases [1]. Oxidized low-density lipoprotein (ox-LDL) acts on endothelial cells (ECs), macrophages, platelets, smooth muscle cells, fibroblasts, and other cells by targeting lectin-like ox-LDL receptor 1 to promote the pathogenesis of AS [2]. ox-LDL can cause endothelial injury by inducing endothelial cell apoptosis, thus aggravating AS [3]. Alleviating ox-LDL-induced endothelial cell injury is of great significance for preventing AS development. Accordingly, it is important to clarify the mechanism of ox-LDL-induced endothelial cell injury.

Circular RNAs (circRNAs) are stably expressed in eukaryotes and have a covalently closed-loop structure [4,5]. CircRNAs can function as competing endogenous RNAs to adsorb microRNAs (miRNAs) to further modulate the expression of various target genes and ultimately play an important regulatory role in various diseases [5,6]. It is reported that circRNAs are aberrantly expressed in the carotid arteries of a rabbit model of AS established by high-fat feeding [7]. Another study shows that circ_0044073 overexpression facilitates the proliferation, migration, and inflammation of human umbilical vein endothelial cells (HUVECs) and human umbilical vein smooth muscle cells (HUVSMCs) by activating JAK/STAT pathway, suggesting circ_0044073 may participate in the pathogenesis of AS [8]. Additionally, circ_PTPRA is up-regulated in AS patients’ plasma and ox-LDL-induced HUVSMCs, and knocking down circ_PTPRA reduces SP1 expression to suppress cell proliferation and induce cell apoptosis, which indicates that circ_PTPRA overexpression can accelerate AS progression [9]. Circ_0033596 is formed by reverse splicing of phosphofurin acidic cluster sorting protein 2 (PACS2) transcript. Interestingly, PACS2 expression is enhanced in ox-LDL-treated HUVECs, and PACS2 overexpression promotes ox-LDL-induced endothelial cell apoptosis [10]. Nonetheless, the biological functions and regulatory mechanisms of circ_0033596 in AS remain unclear.

It is reported that, in ApoE(-/-) mice fed with a high-fat diet, miR-217-5p expression is reduced, and miR-217-5p overexpression represses ox-LDL-induced aortic endothelial cell apoptosis by targeted regulation of the chloride intracellular channel 4 (CLIC4) [11]. Interestingly, our bioinformatics analysis showed that circ_0033596 could probably directly target miR-217-5p. In the present study, we established an *in-vitro* AS model by using ox-LDL to treat HUVECs and to investigate the role of the circ_0033596/miR-217-5p/CLIC4 axis in AS development.

In this study, we hypothesized that circ_0033596 had the potential to be a diagnostic biomarker and therapeutic target for AS. We investigated the biological function and mechanism of circ_0033596 in AS progression. Herein, we found that circ_0033596 promoted ox-LDL-induced HUVEC apoptosis by targeting the miR-217-5p/CLIC4 axis.

## 2. Materials and methods

### 2.1 Cell culture *[12]*

From the American Type Culture Collection (Rockville, MD, USA), HUVECs were obtained. HUVECs were cultured in Dulbecco’s modified Eagle’s medium (DMEM) (HyClone, Logan, UT, USA) with 10% inactivated fetal bovine serum (HyClone, Logan, UT, USA). The cells were placed in 5% CO_2_ in fully saturated humidity at 37°C, and the cells were routinely cultured and passaged once every 2 to 3 days. In subsequent experiments, 100 μg/ml ox-LDL (Sigma-Aldrich, Saint-Louis, MO, USA) was adopted to treat HUVECs for 24 h to construct an AS model.

### 2.2 Cell transfection *[13]*

From GenePharma (Shanghai, China), PACS2 overexpression plasmid (PACS2-oe), circ_0033596 overexpression plasmid (circ_0033596-oe), empty vector (NC), small-interfering RNAs (siRNAs) targeting circ_0033596 (si-circ_0033596#1, si-circ_0033596#2), miR-217-5p mimics and inhibitors, and their negative controls (mim-NC, Inh-NC) were obtained. Following the manufacturer’s instructions, Lipofectamine® 3000 (Invitrogen, Carlsbad, CA, USA) was employed to transfect the above-mentioned vectors/oligonucleotides (50 nM) into HUVECs. Quantitative real-time polymerase chain reaction (qRT-PCR) was utilized to detect the cell transfection efficiency 24 h after the transfection.

### 2.3 qRT-PCR *[14]*

The TRIzol reagent (Invitrogen, Shanghai, China) was employed to extract the total RNA of HUVECs, and then the RNA concentration and purity were measured. First Strand cDNA Synthesis Kit (Thermo Fisher Scientific Inc., Rockford, IL, USA) was used to reverse-transcribe the total RNA into cDNA. With the cDNA as the template, qRT-PCR was performed with a SYBR® Premix-Ex-Taq™ kit (Takara, Tokyo, Japan) in ABI7300 real-time PCR system (Thermo Fisher Scientific, Waltham, MA, USA). GAPDH and U6 were utilized to normalize mRNA expression and miRNA expression, respectively. Below are the specific primer sequences (F: Forward; R: Reverse).

circ_0033596 F: 5′-AGAACCATCCTGGGCTACAA-3′

circ_0033596 R: 5′-CACGGAGATCAGCTCCTTCT-3′

miR-217-5p F: 5′-CGCTACTGCATCAGGAACTGAT-3′

miR-217-5p R: 5′-GTGCAGGGTCCGAGGTATTC-3′

CLIC4 F: 5′-GCAGTGATGGTGAAAGCATAG-3′

CLIC4 R: 5′-TATAAATGGTGGGTGGGTCC-3′

GAPDH F: 5′-GCACCGTCAAGGCTGAGAAC-3′

GAPDH R: 5′-TGGTGAAGACGCCAGTGGA-3′

U6 F: 5′-CTCGCTTCGGCAGCACA-3′

U6 R: 5′-AACGCTTCACGAATTTGCGT-3′

### 2.4 Cell counting kit-8 (CCK-8) assay *[15]*

HUVECs during logarithmic growth were selected, and after trypsinization, and cell density was adjusted to 2 × 10^4^ cells/ml. Next, the cell suspension was added into 96-well plates (100 μL/well). Subsequently, the cells were cultured in the incubator. After 24 h, 10 μL of CCK-8 solution (Biossci, Wuhan, China) was added to each well, and the cells were incubated for another 1 h. After the culturing was terminated, a microplate reader was employed to measure the absorbance at 450 nm wavelength.

### 2.5 Flow cytometry *[16]*

Flow cytometry was carried out to detect HUVEC apoptosis and analyze the cell cycle. The degree of apoptosis was determined using the AnnexinV/7-AAD apoptosis detection kit (Southern Biotechnology, Birmingham, AL, USA). The HUVECs were washed twice with cold phosphate buffer saline (PBS), and re-suspended in binding buffer. Its final concentration was adjusted to 1 × 10^6^ cells/ml, and then the cells were incubated with the AnnexinV-APC staining solution and the 7-AAD staining solution. Eventually, the apoptosis of the HUVECs was determined with a flow cytometer, and Flow Jo V10 software (BD Biosciences, San Diego, CA, USA) was applied to analyze the results. For the cell cycle analysis, the HUVECs were fixed with 70% ethanol, and finally kept overnight at 4°C. After that, the cells were rinsed with PBS, and then, propidium iodide staining solution was added to make a final concentration of 0.05 mg/ml, and the cells were stained at 4°C for 30 min. Ultimately, the cell cycle was analyzed with a flow cytometer.

### 2.6 RNA immunoprecipitation (RIP) assay *[17]*

A Magna RIP™ RNA-binding protein immunoprecipitation kit (Millipore, Billerica, MA) was utilized to conduct the RIP assay. HUVECs during logarithmic growth were harvested and suspended in a RIP lysis buffer to prepare cell lysate. Subsequently, the cell lysate was incubated with an anti-Ago2 antibody or control IgG coupled with magnetic beads, followed by the purification of RNA. The purified RNA was reverse-transcribed into cDNA. Ultimately, circ_0033596 and miR-217-5p expression were detected via qRT-PCR.

### 2.7 Dual-luciferase reporter gene assay *[18]*

To construct wild-type (circ_0033596 WT, CLIC4 WT) or mutant (circ_0033596 MUT, CLIC4 MUT) luciferase reporter gene vectors, we amplified the binding sequences predicted by bioinformatics between miR-217-5p and circ_0033596 or between miR-217-5p and CLIC4 mRNA 3’-UTR or the mutated sequences, and cloned them into the pmirGLO Dual-Luciferase miRNA Target Expression Vector (Promega, Madison, WI, USA). The above-mentioned reporter vectors and miR-217-5p mimics (or the negative control) were co-transfected into HUVECs. The luciferase activity was measured with a dual-luciferase reporter gene detection kit (Promega, Madison, WI, USA) 48 h after the transfection.

### 2.8 Western blot assay *[19]*

The medium was discarded, and the HUVECs were lysed in a pre-cooled RIPA lysis buffer (Beyotime Biotechnology, Shanghai, China) on ice for 20 min, and the supernatant was harvested after centrifugation for 20 min. Then, the total protein of cells was extracted. 20 μg of total protein in each sample was separated by electrophoresis. Next, the protein was electro-transferred to a polyvinylidene fluoride (PVDF) membrane. The PVDF membrane was blocked with 5% skimmed milk for 1 h at room temperature, washed 3 times with tris buffered saline tween (TBST), 10 min for each time, and then incubated with the primary antibody (anti-CLIC4 rabbit polyclonal antibody, ab153836, Abcam, Shanghai, China, 1:1000 dilution) at 4°C overnight. After the membrane was washed with TBST, the membrane and the secondary antibody (goat anti-rabbit IgG, ab205718, Abcam, Shanghai, China, 1:2000 dilution) were incubated for 1 h at room temperature. Next, the membrane was washed 3 times with TBST, 10 min for each time. Eventually, an enhanced chemiluminescence kit (Amersham Pharmacia Biotech, Little Chalfont, UK) was employed to develop the protein bands.

### 2.9 Statistical analysis *[20]*

In the present study, all of the experiments were performed in triplicate. The tool for statistical analysis was SPSS 23.0 statistical analysis software (SPSS Inc., Chicago, IL, USA). A comparison of the mean values between the two groups was conducted using Student’s *t*-test, and a comparison of the mean values among multiple groups was conducted employing a one-way analysis of variance. *P* < 0.05 served as the criterion for significant difference.

## 3. Results

We hypothesized that circ_0033596 could promote the progression of AS via modulating the endothelial injury. Gain-of-function and loss-of-function models have been established in the present work, and we report that circ_0033596 regulates ox-LDL-induced HUVEC apoptosis *in vitro*. Additionally, it is revealed that circ_0033596 directly targets miR-217-5p and up-regulates CLIC4 expression, thus promoting ox-LDL-induced HUVEC apoptosis.

### 3.1 Circ_0033596 is up-regulated in ox-LDL-induced HUVECs

**Figure 1.**
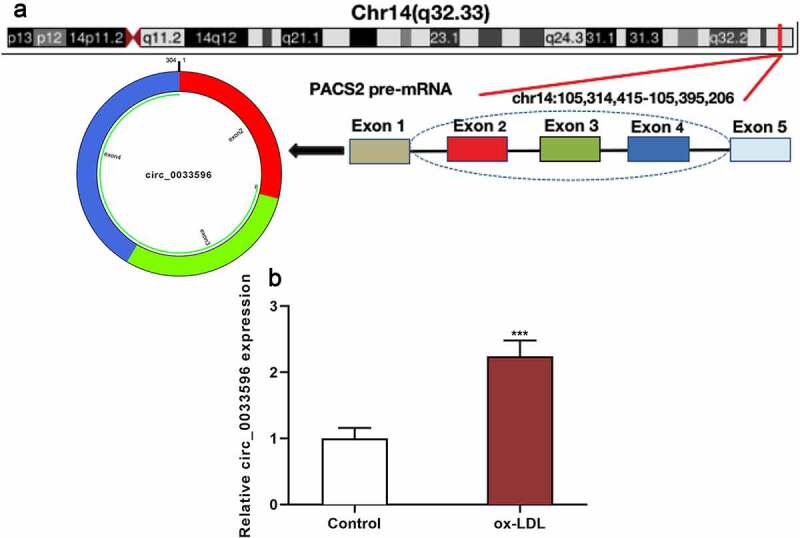
Circ_0033596 is up-regulated in HUVECs treated with ox-LDL.

PACS2 is involved in ox-LDL-induced injury of endothelial cells [10], and the circBase database showed that circ_0033596 is derived from the reverse splicing of PACS2 transcript (Figure 1a). Subsequently, the HUVECs were treated with 100 μg/ml ox-LDL and circ_0033596 expression was detected via qRT-PCR, and it was revealed that circ_0033596 expression was elevated in ox-LDL-treated HUVECs (Figure 1b).

(a) The generation process of circ_0033596 was queried from circBase database, and a diagram was drawn. (b) HUVECs were treated with 100 μg/ml ox-LDL, and qRT-PCR was conducted to detect the expression of circ_0033596 in the treated cells and untreated cells.***P < 0.001.

### 3.2 Effects of circ_0033596 overexpression or knockdown on HUVEC viability, cell cycle progression, and apoptosis

**Figure 2.**
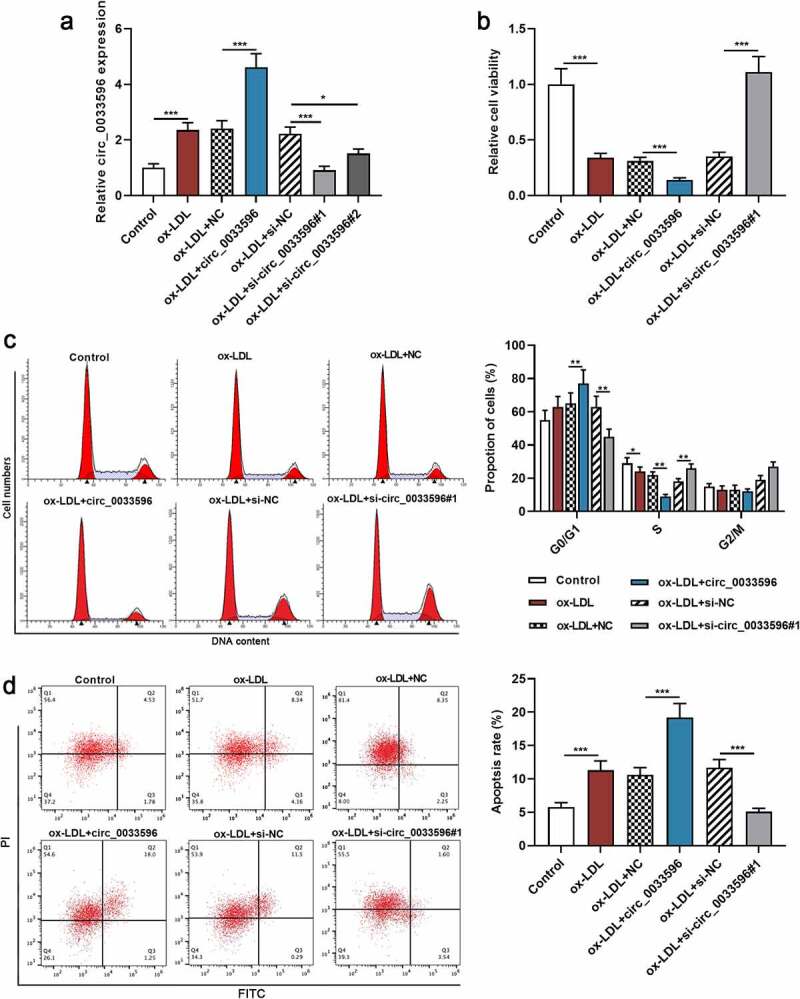
Effects of circ_0033596 overexpression or knockdown on HUVEC viability, cell cycle progression and apoptosis.

To delve into the biological functions of circ_0033596 in AS, HUVECs were transfected with circ_0033596 overexpression plasmid and si-circ_0033596#1 or si-circ_0033596#2, and the transfection was shown by qRT-PCR to be successful (Figure 2a, Supplementary Figure 1A). Subsequently, CCK-8 assay and flow cytometry were performed to detect the effects of circ_0033596 on the viability, cell cycle progression, and apoptosis of ox-LDL-induced HUVECs. The results showed that ox-LDL treatment markedly restrained cell viability, blocked cell cycle progression, and facilitated apoptosis, and circ_0033596 overexpression aggravated these effects; knocking down circ_0033596 promoted cell viability, accelerated cell cycle progression, and inhibited cell apoptosis (Figure 2b-d). Notably, there was no significant difference between the effects of circ_0033596 overexpression and PACS2 overexpression on apoptosis of HUVECs (Supplementary Figure 1B).

(a) Circ_0033596 overexpression plasmid or si-circ_0033596#1/2 was transfected into ox-LDL-induced HUVECs, and qRT-PCR was employed to detect circ_0033596 expression to verify the transfection efficiency. (b) HUVEC viability was detected by CCK-8 assay after the transfection. (c) The cell cycle progression of HUVECs was detected via flow cytometry after the transfection. (d) Flow cytometry was utilized to detect HUVEC apoptosis after the transfection.*P < 0.05, **P < 0.01, and ***P < 0.001.

### 3.3 Circ_0033596 directly targets miR-217-5p

**Figure 3.**
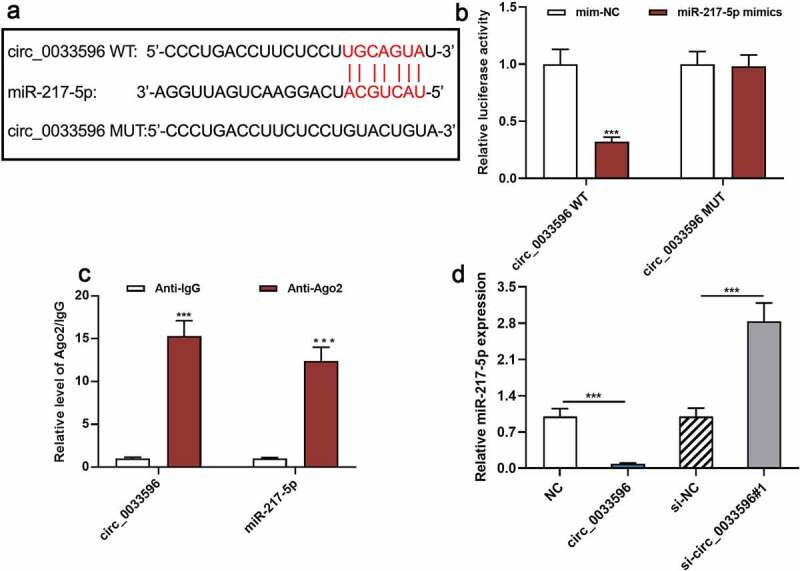
Targeted relationship between circ_0033596 and miR-217-5p.

**Figure 4.**
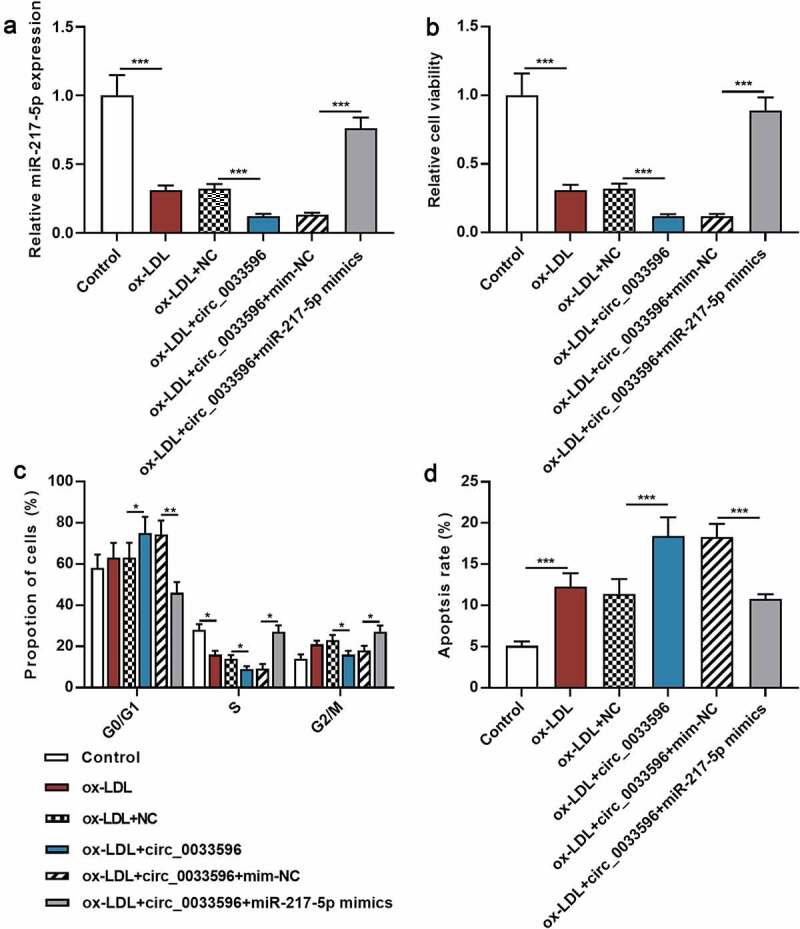
Circ_0033596 modulates the viability, cell cycle progression and apoptosis of HUVECs via targeting miR-217-5p.

To clarify the downstream mechanism of circ_0033596, miR-217-5p was found to be a potential downstream target of circ_0033596 through searching the StarBase database (starbase.sysu.edu.cn) (Figure 4a). The dual-luciferase reporter gene assay indicated that the transfection of miR-217-5p mimics remarkably suppressed the luciferase activity of the circ_0033596 WT reporter yet failed to significantly affect that of circ_0033596 MUT (Figure 4b). Additionally, the RIP assay confirmed that compared with the control group, circ_0033596 and miR-217-5p were significantly enriched in Ago2-containing microribonucleoproteins (Figure 3c). Additionally, qRT-PCR suggested that circ_0033596 overexpression notably repressed miR-217-5p expression, whereas circ_0033596 knockdown facilitated miR-217-5p expression in HUVECs (Figure 3d).

(a) The StarBase database was adopted to predict the binding site between circ_0033596 and miR-217-5p. (b) Dual-luciferase reporter gene assay was conducted to verify the effects of miR-217-5p overexpression on the luciferase activity of circ_0033596 WT and circ_0033596 MUT. (c) RIP assay was performed to further verify the binding relationship between circ_0033596 and miR-217-5p. (d) qRT-PCR was used to detect the effect of circ_0033596 overexpression or knockdown on miR-217-5p expression in HUVECs.***P < 0.001.

### 3.4 Circ_0033596 promotes HUVEC injury through adsorbing miR-217-5p

To further delve into the regulatory effects of circ_0033596 and miR-217-5p on HUVEC apoptosis, circ_0033596 overexpression plasmids and miR-217-5p mimics were co-transfected into ox-LDL-induced HUVECs, and the transfection was confirmed by qRT-PCR to be successful (Figure 4a). Subsequently, CCK-8 assay and flow cytometry were utilized to detect the effects of circ_0033596 and miR-217-5p on ox-LDL-induced HUVEC viability, cell cycle progression, and apoptosis. It was revealed that ox-LDL treatment significantly inhibited cell viability, arrested cell cycle, and promoted apoptosis, and circ_0033596 overexpression aggravated these effects; the transfection of miR-217-5p mimics enhanced cell viability, accelerated cell cycle progression, and suppressed cell apoptosis (Figure 4b-d). Additionally, CCK-8 assay showed that the transfection of miR-217-5p inhibitors inhibited the viability of HUVECs (Supplementary Figure 1C).

(a) Ox-LDL-induced HUVECs were transfected with circ_0033596 overexpression plasmids and miR-217-5p mimics, and qRT-PCR was utilized to detect miR-217-5p expression to verify the transfection efficiency. (b) Circ_0033596 overexpression plasmids and miR-217-5p mimics were transfected into ox-LDL-induced HUVECs, and the cell viability was detected using CCK-8 assay. (c) The cell cycle progression of HUVECs was detected through flow cytometry after the transfection. (d) Flow cytometry was employed to detect the apoptosis of HUVECs after the transfection.*P < 0.05, **P < 0.01 and ***P < 0.001.

### 3.5 Circ_0033596 increases CLIC4 expression by adsorbing miR-217-5p

**Figure 5.**
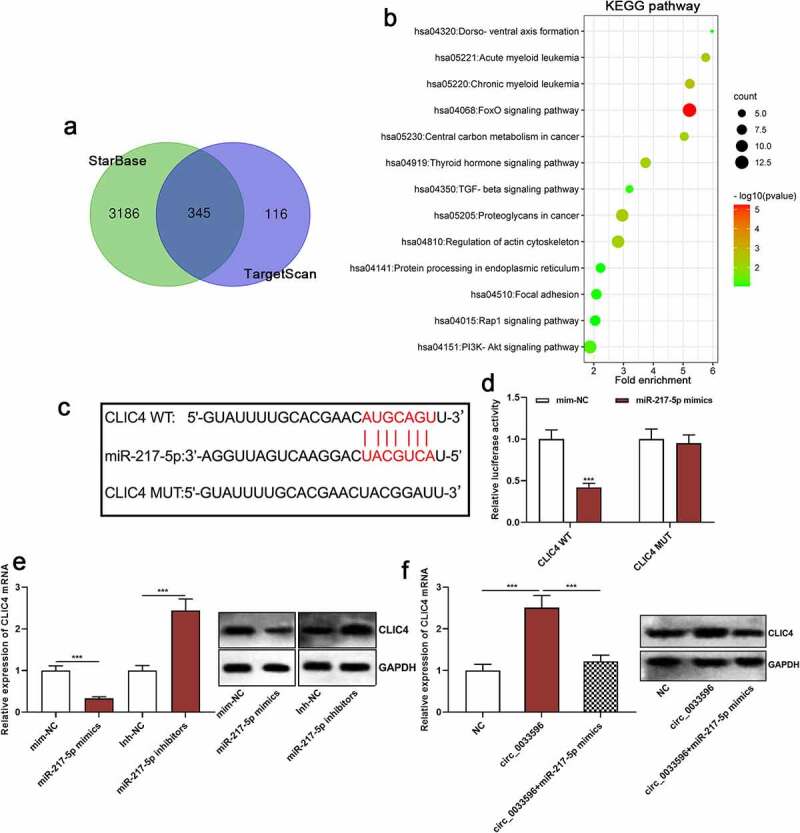
Circ_0033596 up-regulates CLIC4 expression by adsorbing miR-217-5p.

Next, the StarBase database and TargetScan database (http://www.targetscan.org/) were utilized to predict the downstream target genes of miR-217-5p, and it was revealed that there is a total of 345 candidate targets (Figure 5a). Then, the Kyoto Encyclopedia of Genes and Genomes (KEGG) database was applied to analyze the signaling pathways in which the above-mentioned target genes are enriched, and it was indicated that the target genes of miR-217-5p were mainly enriched in the transforming growth factor-beta (TGF-beta) signaling pathway and the PI3K/Akt/mTOR pathway, which were closely related to AS pathogenesis (Figure 5b). Among them, CLIC4, which is associated with AS development, was one of the candidate targets of miR-217-5p, and its binding sequence is shown in Figure 5c. The dual-luciferase reporter gene assay confirmed that miR-217-5p overexpression markedly repressed the luciferase activity of the CLIC4 WT reporter but failed to significantly affect that of CLIC4 MUT (Figure 5d). After that, qRT-PCR was conducted to detect the effects of transfection with miR-217-5p mimics or inhibitors on CLIC4 mRNA and protein expression, and it was revealed that the miR-217-5p overexpression inhibited CLIC4 mRNA and protein expression, whereas inhibition of miR-217-5p promoted CLIC4 mRNA and protein expression (Figure 5e). These results suggested that CLIC4 was a target gene of miR-217-5p, which is consistent with the previous report [11]. Furthermore, we demonstrated that circ_0033596 overexpression promoted CLIC4 mRNA and protein expression and the transfection of miR-217-5p mimics weakened this effect (figure 5f). In addition, HUVECs were treated with 100 μg/ml ox-LDL and CLIC4 mRNA expression was detected via qRT-PCR, and it was revealed that CLIC4 mRNA expression was elevated in ox-LDL-treated HUVECs (Supplementary Figure 1D).

(a) The StarBase and TargetScan databases were utilized to predict the downstream target genes of miR-217-5p, and the Venn diagram was applied to display the number of target genes. (b) The KEGG database was adopted to perform a pathway enrichment analysis of the target genes of miR-217-5p. (c) Binding sites of miR-217-5p with CLIC4 WT and CLIC4 MUT luciferase reporters. (d) Dual-luciferase reporter gene assay was used to detect the effects of miR-217-5p overexpression on the luciferase activity of CLIC4 WT and CLIC4 MUT. (e) qRT-PCR and Western blot were conducted to detect the effects of transfection with miR-217-5p mimics or inhibitors on CLIC4 mRNA and protein expression in HUVECs. (f) qRT-PCR and Western blot were utilized to detect the effects of circ_0033596 and miR-217-5p on CLIC4 mRNA and protein expression in HUVECs.***P < 0.001.

## 4. Discussion

AS is the main cause of cardiovascular diseases; the onset of AS is currently believed to be related to hyperlipidemia, especially the dysregulation of ox-LDL [21]. ox-LDL can cause the increase of pro-inflammatory cytokines and adhesion molecules, activate the apoptosis pathway, and thereby cause endothelial cell dysfunction, which promotes AS progression [2]. In the last few years, some circRNAs have been reported to be abnormally expressed in AS and are associated with the injury of vascular endothelial cells induced by ox-LDL [22–24]. For instance, circ_0003645 silencing can reduce ox-LDL-induced HUVEC apoptosis and inflammation through regulating NF-κB pathway [23]. circ_0001879 promotes the progression of AS by regulating the proliferation and migration of ox-LDL-induced HUVECs by up-regulating histone deacetylase 9 expression [24]. Another study reports that circHIPK3 is down-regulated in AS mice and ox-LDL-treated HUVECs; circHIPK3 can reduce the lipid level in ox-LDL-induced HUVECs by activating autophagy [25]. In addition, circ_0124644 modulates pregnancy-associated plasma protein-A via sponging miR-149-5p, thereby aggravating ox-LDL-induced HUVEC injury [26]. In the present study, for the first time, we demonstrate that circ_0033596 expression is reduced in ox-LDL-induced HUVECs, and circ_0033596 overexpression remarkably represses ox-LDL-induced injury of HUVECs. These data suggest that circ_0033596 is a crucial regulator in AS pathogenesis, especially in the process of vascular endothelial cell injury.

Known as a class of endogenous non-coding RNAs, microRNAs (miRNAs) contain 18–25 nucleotides; they are highly conserved and can bind with messenger RNA (mRNA) 3’-untranslated region (3’-UTR) to regulate translation process and participate in many biological processes [27]. MiRNAs, as important regulators of cell adhesion, proliferation, apoptosis, lipid efflux, and the production of inflammatory mediators, play an important role in AS pathogenesis. For instance, miR-21 is down-regulated in serum samples of AS patients and AS plaques of ApoE(-/-) mice, and miR-21 restrains vascular smooth muscle cell proliferation through suppressing PTEN, blocking the development of AS [28]. Another study reveals that in the mouse model of AS, neutrophil microvesicles aggregate in vulnerable parts of arteries, and neutrophils deliver miR-155-carrying microvesicles to lesions to enhance NF-κB activation, thus leading to vascular inflammation [29]. MiR-217-5p has been confirmed to be closely related to AS; miR-217-5p is up-regulated in ox-LDL-treated HUVECs, and miR-217-5p modulates the expression of apoptosis-related genes by targeting CLIC4 and reduces mitochondrial membrane permeability, thus suppressing ox-LDL-induced HUVEC injury induced by ox-LDL [11]. The present study demonstrates that circ_0033596 directly targets miR-217-5p and negatively modulates its expression in HUVECs, and the miR-217-5p restoration can reverse the promoting effect of circ_0033596 on HUVEC injury. These findings indicate that the circ_0033596/miR-217-5p axis can aggravate ox-LDL-induced HUVEC injury to promote the development of AS.

The CLIC4 gene is located on chromosome 1p36.1 and codes the CLIC4 protein [30]. CLIC4 is expressed in different organelles or sub-cellular structures such as cell nucleus and microfilament, and the activity of CLIC4 depends on its redox state [31,32]. The role of CLIC4 in human vascular endothelial cell apoptosis and injury has been reported. Specifically, CLIC4 overexpression promotes the accumulation of MDA in ox-LDL-induced cerebral microvascular endothelial cells, reduces the activity of SOD and CAT, and facilitates the apoptosis [33]. Another study reports that CLIC4 is up-regulated in ox-LDL-induced HUVECs, and down-regulating CLIC4 increases ox-LDL-induced HUVEC viability, promotes cell cycle progression and angiogenesis, and reduces apoptosis, inflammation and oxidative stress; this study also reports that CLIC4 is targeted by miR-599 and its expression is negatively regulated by miR-599 [34]. Notably, miR-217-5p directly targets CLIC4 to reduce ox-LDL-induced HUVEC injury [11]. In this work, our data support that circ_0033596 is the upstream regulator of the miR-217-5p/CLIC4 axis, partly explains the mechanism of miR-217-5p/CLIC4 axis dysregulation in vascular endothelial cells stimulated by ox-LDL.

## 5. Conclusion

To sum up, through a series of *in-vitro* experiments, the present study confirms that circ_0033596 aggravates ox-LDL-induced HUVEC injury through regulating miR-217-5p/CLIC4 axis. Our study demonstrates that circ_0033596 may partake in vascular endothelial cell damage during AS development and may become a potential target for AS treatment.

## Highlights

Circ_0033596 expression was increased in ox-LDL-induced HUVECs

Overexpression of circ_0033596 promoted ox-LDL-induced apoptosis of HUVECs.

Circ_0033596 negatively regulated miR-217-5p and positively regulated CLIC4 expression.

Circ_0033596 promoted the injury of endothelial cells to participate in the pathogenesis of atherosclerosis.
